# Integrative genomic and functional characterization of halotolerant *Bacillus paralicheniformis* MHN12 for sustainable agriculture

**DOI:** 10.3389/fmicb.2025.1736288

**Published:** 2026-01-23

**Authors:** Priyanka Dahiya, Shruti Dhiman, Pradeep Kumar, Simran Rani, A. Sankara Narayanan, Kiran Arora, Amita Suneja Dang, Pooja Suneja

**Affiliations:** 1 Plant-Microbe Interaction Laboratory, Department of Microbiology, Maharshi Dayanand University, Rohtak, India; 2 Department of Life Sciences, Sri Sathya Sai University for Human Excellence, Navanihal, Kalaburagi, India; 3 Department of Chemistry, Kirori Mal College, University of Delhi, New Delhi, India; 4 Centre for Medical Biotechnology, Maharshi Dayanand University, Rohtak, India

**Keywords:** Bacillus paralicheniformis, biosynthetic gene clusters, halotolerant bioinoculant, plant growth promoting endophyte, sustainable agriculture, whole-genome

## Abstract

**Introduction:**

This study clarifies the taxonomic identity of *Bacillus paralicheniformis* MHN12 and maps the genetic foundations of its beneficial traits. It also provides functional insights into the salinity-stress response and paves the way for the development of MHN12 as a potential bioinoculant to enhance crop stress resilience and productivity

**Methods:**

The endophytic strain MHN12, isolated from *Vigna radiata*, was initially identified as *Bacillus licheniformis* based on its 16S rRNA sequence. To ascertain its identity and ensure accurate taxonomic classification, a comparative genomic analysis based on genome relatedness indexes and secondary metabolite biosynthetic gene clusters was conducted, involving MHN12 and 22 other *B. paralicheniformis* strains.

**Results and discussion:**

There were high similarities among the strains and antiSMASH revealed the presence of biosynthetic gene clusters specifically fengycin and bacitracin in MHN12 encoded by the genomes of *B. paralicheniformis* but absent in *B. licheniformis*. The whole genome analysis of *B. paralicheniformis* MHN12, focusing on identifying genes contributing to its potential to promote plant growth and abiotic stress tolerance was also performed. Genes linked to chemotaxis, motility, polysaccharide synthesis, plant growth promoting traits, antimicrobial and stress mitigation compounds were annotated. This highlights MHN12's potential to efficiently colonize plants, stimulate their growth, and protect them from environmental stresses and pathogens. *In vitro* assays also supported the genomic data, demonstrating MHN12's ability to synthesize enzymatic antioxidants and exopolysaccharides (EPS) while retaining plant growth promoting traits under salinity stress. Gas chromatography (GC)-based analysis revealed modulation of plasma membrane lipids aiding MHN12 to combat salt stress.

## Graphical Abstract

**Figure:**
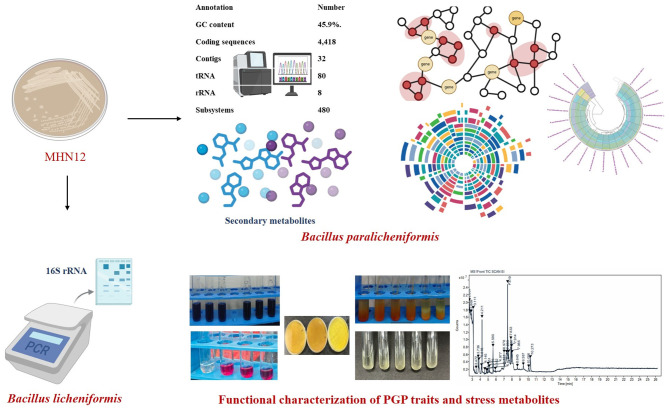

## 1 Introduction

The world's population is projected to reach 9.3 billion by 2050, demanding a 60% increase in food production to ensure global food security (Fischer et al., 2014). This necessity must be met sustainably, as traditional agricultural practices relying on the overuse of chemical fertilizers and pesticides degrade soil health, harm beneficial microbial diversity, and cause environmental problems. Climate change further intensifies agricultural challenges with biotic and abiotic stresses like drought, salinity, temperature extremes, and plant pathogens (Mishra et al., 2021; Rajkumar et al., 2024). Salinity stress is a particular concern, affecting over 1 billion hectares of land globally and hampering agricultural productivity (Mishra et al., 2021). Furthermore, limited land resources and declining biodiversity complicate the achievement of demand vs. supply of food for rising population (Tilman et al., 2017). To sustainably improve agricultural production, and address these complex, intertwined challenges along with the impacts of climate change, researchers are looking for eco-friendly alternatives to conventional agrochemicals.

Plant science and biotechnology advancements offer promising strategies to counter these challenges and the application of plant growth-promoting endophytic bacteria (PGPEB) is one promising sustainable approach (Srikanth et al., 2025). By colonizing plant tissues, PGPEB boosts plant growth via phytohormone synthesis, nutrient acquisition, and producing antimicrobial metabolites. PGPEB exhibits stress resilience by accumulating compatible solutes, inducing ion channels, and upregulating antioxidant machinery (Dudeja et al., 2021; Rani et al., 2022). Studies exploring the full potential of PGPEB via a deeper understanding of the pathways and genes responsible for their beneficial and stress management traits are increasing significantly. This positions PGPEB as a valuable tool for enhancing agricultural productivity under stress conditions (Vurukonda et al., 2016; Chen et al., 2019; Kumar et al., 2020; Kushwaha et al., 2020; Bhutani et al., 2021b). Genome sequencing has emerged as a powerful tool, providing insights into beneficial microorganisms. Genome sequencing and analysis by aiding the identification and categorization of genes reveal beneficial microorganisms' adaptation strategies, metabolic and functional features for promoting plant growth and stress resilience. However, draft genomes with inaccuracies and low completeness limit comprehensive analysis in areas like phylogenomics and pan-genomics. Therefore, generating high-quality whole genome sequencing (WGS) data is essential to realize the potential of these beneficial microorganisms fully (Sharma et al., 2023). Recent studies (Chaudhary et al., 2021; Gao et al., 2022; Kumar et al., 2022) have revealed that WGS can provide abundant genomic data that can be mined for potential agricultural applications. The high-quality, well-assembled genomes are critical for robust and reliable analyses. Comparative genome analyses also have some limitations due to low-quality or incomplete assemblies, where significant gaps could lead to inaccurate gene prediction and reduce confidence in strain-level comparisons (Alkan et al., 2011).

*Bacillus*, a Gram-positive Firmicute, is well known for its excellent secretion systems, and production of an array of extracellular enzymes like amylases, cellulases, proteases, pectinases, etc. This makes *Bacillus* spp. vital in diverse sectors such as detergent, textile, food, feed, and beverage (Sansinenea, 2019; Bhutani et al., 2021a,b). Many past identifications grouped *Bacillus paralicheniformis* under *Bacillus licheniformis* due to their close relatedness. A recent genomic study revealed secondary metabolite operons i.e., fengycin and bacitracin specific to *B. paralicheniformis* only but absent in *B. licheniformis* (Olajide et al., 2021). This taxonomic clarification highlights a critical knowledge gap regarding its potential in promoting plant growth as evidenced by 232 publications listed in the Scopus database as of 12th May, 2025. The existing literature predominantly explores *B. paralicheniformis* for industrial and biotechnological applications, with limited attention on its potential contribution to sustainable agriculture as plant growth-promoting bacteria (Supplementary Figure 1). In previous studies, *B. paralicheniformis* strain MHN12 was isolated from the nodules of *Vigna radiata*. Based on 16S rRNA sequence similarity initially, the isolate was submitted as *B. licheniformis* (Bhutani et al., 2021b). The strain possessed antimicrobial activity, plant growth promotion abilities, and high salinity tolerance that indicate its role in supporting plant growth and biocontrol (Bhutani et al., 2022). In the current study, whole-genome sequencing of strain MHN12 was done followed by a comparative analysis with other *B. paralicheniformis* strains to ensure accurate taxonomic identification. To assess its potential for commercialization as a bioinoculant, a detailed genome analysis was performed. Genetic determinants and metabolic pathways responsible for the biosynthesis of biomolecules associated with plant growth promotion and stress tolerance in strain MHN12 were identified. *In vitro* potential of strain MHN12 to synthesize these compounds under saline stress conditions was also assessed. Furthermore, Gas Chromatography-Mass Spectrometry (GC-MS) was performed to compare its metabolite profiles under control and stress conditions.

## 2 Methods

### 2.1 Bacterial sample and genome sequencing

The *Bacillus paralicheniformis* strain MHN12 used in this study was originally isolated from nodules of *Vigna radiata* and is maintained in Lab 312, Department of Microbiology, Maharshi Dayanand University, Rohtak. The isolate was grown on a Tryptic Soy Agar (TSA) medium and its purity was confirmed by Gram staining. The bacterial colony was sent to Eurofins Genomics India Pvt. Ltd for sequencing. DNA was extracted using the Quick-DNA Miniprep Plus kit (Zymo Research). Further, it was quantified and assessed for quality by NanoDrop Spectrophotometer. A paired-end sequencing library from genomic DNA was prepared using the Illumina TruSeq Nano DNA Library Prep kit and subsequently sequenced on the Illumina NextSeq500 platform. Quality control of the raw sequencing reads was done using FastQC and adapter sequences, low-quality reads were filtered and trimmed using Fastp (Andrews, 2010; Chen et al., 2018). The obtained filtered reads were used for *de novo* genome assembly by Shovill v1.0.4 (Seemann, 2014). Finally, the assembled genome was identified and annotated using Galaxy (https://usegalaxy.org/) and Rapid Annotation Subsystem Technology (RAST) (https://rast.nmpdr.org/).

### 2.2 Strain selection and phylogenomic metric calculations

A total of 22 *B. paralicheniformis* strains with comprehensive characterization in the literature and complete genome data available in the NCBI database were selected for genomic comparison with our in-house strain MHN12. Genome and protein sequences were retrieved from the NCBI (https://www.ncbi.nlm.nih.gov/). To assess the relatedness and taxonomic classification, three methods were employed: Digital DNA–DNA Hybridization (dDDH), Average Nucleotide Identity (ANI), and Average Amino acid Identity (AAI). The Genome-to-Genome Distance Calculator (GGDC 2.0) webserver was utilized to calculate dDDH values, applying formula 2 (Meier-Kolthoff et al., 2013). The enveomics package with the two-way ANI and AAI options was used to calculate ANI and AAI values (Rodriguez-R and Konstantinidis, 2016). The obtained values were used to generate matrices and subsequently visualized on a heatmap.

### 2.3 Genome mining and core and pan-genome analysis

Core and pan-genome profiles were generated using the Bacterial Pan Genome Analysis Pipeline (BPGA) (Chaudhari et al., 2016). Functional genes derived from the pan-genome and the core genome were assigned Kyoto Encyclopedia of Genes and Genomes (KEGG) categories using the USEARCH program query of the KEGG database within the BPGA pipeline. A pairwise genomic alignment of all 23 *B. paralicheniformis* strains was created using BRIG 0.95 (Alikhan, 2011). To analyze genome rearrangements and alignments, *B. paralicheniformis* strain MHN12 was compared with strain FA6 by Mauve v 2.3.1 (Darling et al., 2004). The secondary metabolite biosynthetic gene clusters within the genomes of 23 *B. paralicheniformis* strains were identified using the Antibiotic and Secondary Metabolite Analysis Accessory (AntiSMASH) online server v7.0 (Blin et al., 2023).

### 2.4 Functional pathway analysis and genome annotation

The Figure IDs acquired via RAST annotation for strain MHN12 strain were used to reconstruct metabolic pathways by the MinPath server (version 1.2). The MinPath server employs a parsimony approach that utilizes protein family predictions to reconstruct a conservative set of biological pathways for a query genome (Ye and Doak, 2009). The derived pathways were subsequently sorted focusing on plant growth-promoting and stress-resilience traits. For gene annotation of strain MHN12 web-based server BlastKOALA was employed (Kanehisa et al., 2016). The resulting annotations and functional predictions were manually evaluated against existing literature.

### 2.5 Characterization of multi-PGP traits and stress specific metabolites under salinity stress

The plant growth-promoting (PGP) traits including indole acetic acid (IAA) production, ammonia and hydrogen cyanide (HCN) production, and phosphate solubilization, were assessed under control conditions and under salt stress (10% NaCl) (Bhutani et al., 2021b,a). To evaluate its stress-alleviating potential, the production of enzymatic antioxidants superoxide dismutase (SOD) and catalase (CAT), as well as exopolysaccharides (EPS), under the same saline conditions were quantified (Gao et al., 1998; Aebi, 1984; Silambarasan et al., 2019).

### 2.6 Extraction and characterization of metabolites from MHN12 under salinity stress

The strain MHN12 was inoculated into TSB with NaCl (10%) and without NaCl (control). Following incubation of 24 h, the bacterial cells were pelleted by centrifugation at 10,000 × g for 10 mins, and the cell-free supernatant was collected. The supernatant was acidified to pH 4.0 and extracted with an equal volume of ethyl acetate. The organic phase was collected and dried in a rotary evaporator (Arun et al., 2020). The resulting extract was re-dissolved in HPLC grade methanol for subsequent analysis. The samples for Chromatography–Mass Spectrometry (GC–MS) were sent to a service provider. The resulting mass spectra were compared against the NIST (National Institute of Standards and Technology) version 2.3 mass spectral library for compound identification and those related to salinity mitigation were focused.

## 3 Results

### 3.1 Sequencing metrics

The complete genome of *Bacillus paralicheniformis* strain MHN12 was sequenced on Illumina NextSeq500 technology, achieving approximately 100x coverage. After filtering for low-quality reads, 5,043,632 paired-end reads were generated. The reads were assembled, and the total length of 4,245,453 bp with N50 of 503,829 bp was generated. The final assembly comprised 32 contigs and 4,418 coding sequences (CDS).

### 3.2 Genomic characteristics of MHN12 and phylogenomic analysis

**Figure 1:**
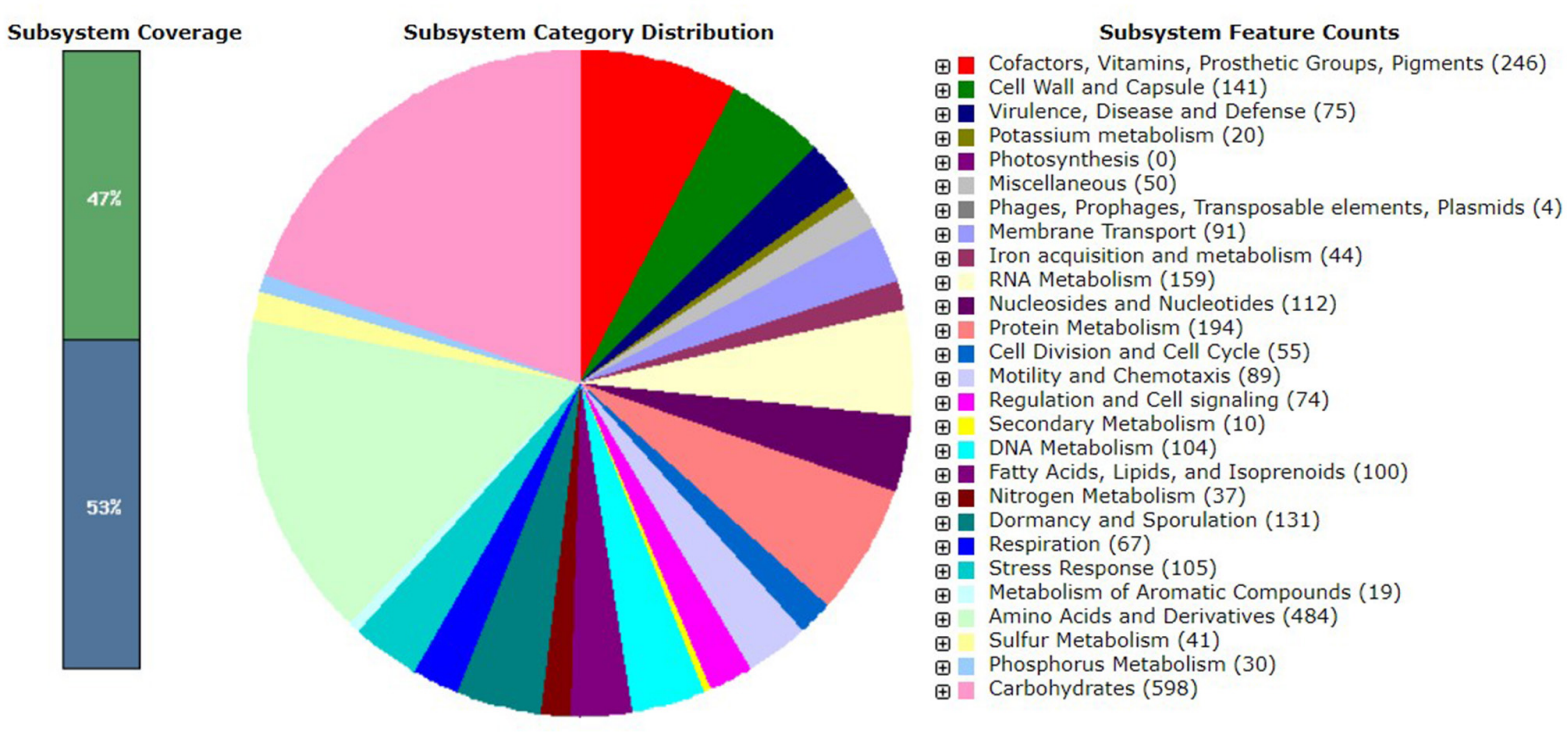
Subsystem analysis *of bacillus paralicheniformis* MHN12 genome performed using the RAST annotation tool.

**Figure 2:**
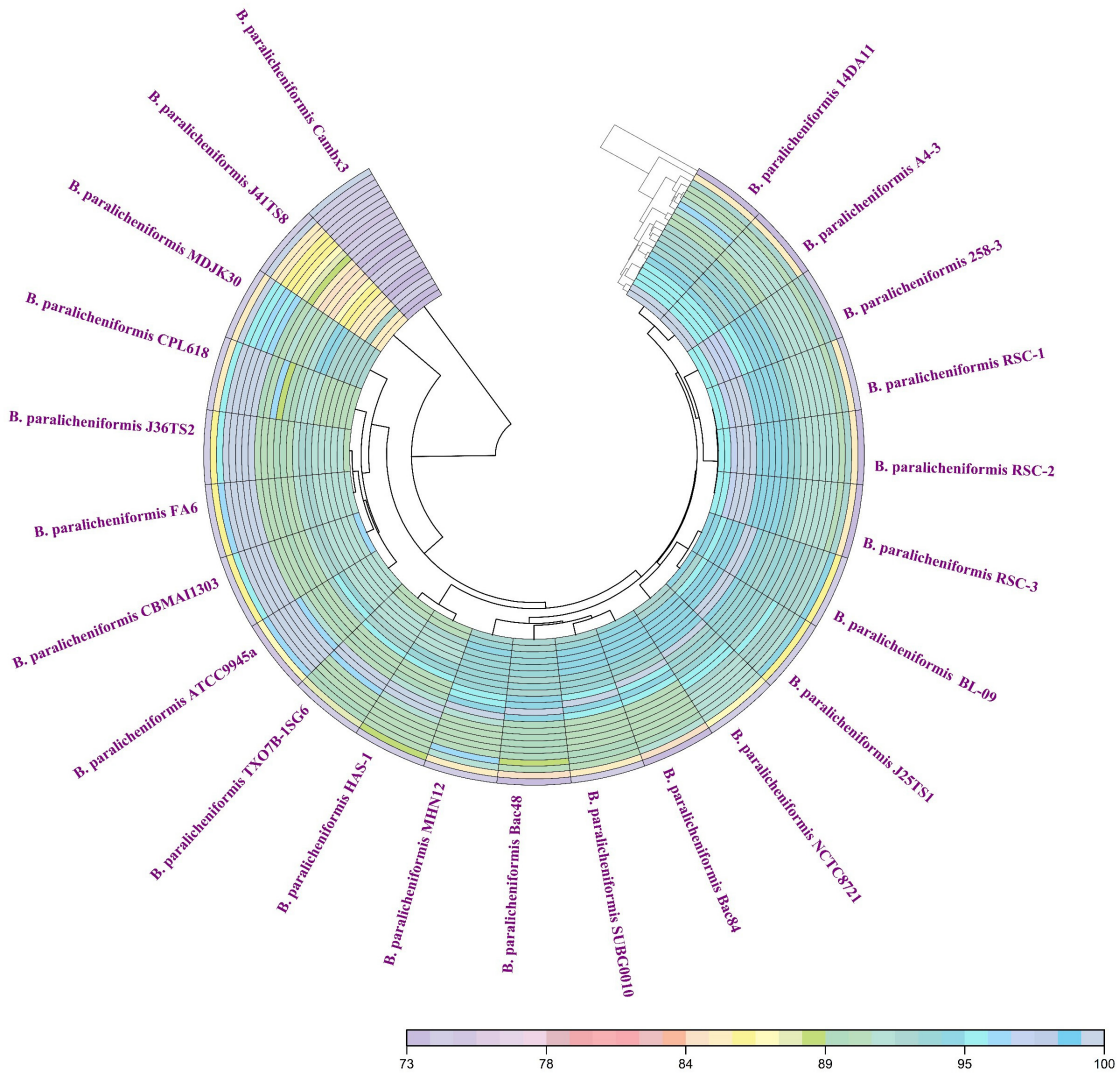
Heat map illustrating digital DNA–DNA hybridization (dDDH) between the strains under study.

**Figure 3:**
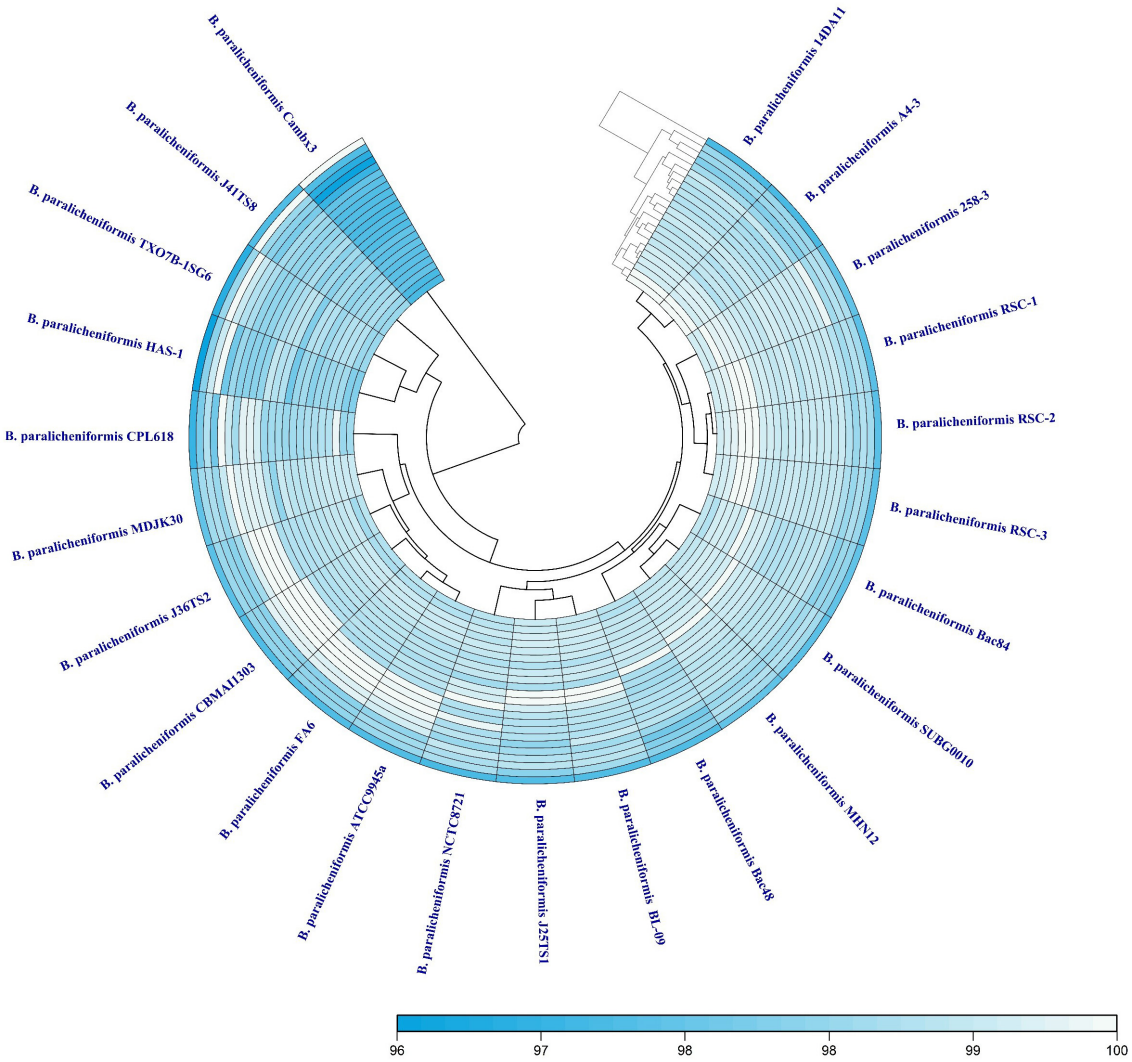
Heat map illustrating average amino acid identity (AAI) between the strains under study.

Members of the genus *Bacillus*, including *B. paralicheniformis*, typically possess a circular chromosome of ~4.2–4.3 Mbp. The strain MHN12 draft genome assembled into 32 contigs has an estimated size of ~4.24 Mbp and a GC content of 45.9%. It contained 80 tRNA and 8 rRNA genes. Analysis of strain MHN12 using RAST identified 480 subsystems encompassing diverse metabolic (carbohydrates, amino acids, cofactors, vitamins, prosthetic groups) and cellular functions (pigmentation, stress response, iron metabolism, motility, chemotaxis) essential for the organism's survival and function (Figure 1) For comparative analysis, the features of selected *B. paralicheniformis* strains were compiled using NCBI datasets and scientific literature, summarized in Table 1. While the GC content and number of coding sequences of strain MHN12 resembled other strains, its genome size was smaller. *B. paralicheniformis* J36TS2 and A4-3 had larger genomes (4.6 Mbp and 4.58 Mbp respectively). Notably, many strains exhibit potential for biocontrol, with some (including strain MHN12 and MDJK30) known for plant growth promotion, and FA6 for probiotic applications. The Digital DNA–DNA Hybridization (dDDH), Average Nucleotide Identity (ANI), and Average Amino acid Identity (AAI) analyses revealed high genomic and proteomic similarities among the strains. All strains ' dDDH values exceeded 70%, with the lowest value (72.5%) observed between CamBx3 and 14AD11 (Figure 2). Most strains had ANI and AAI values exceeding 95%, suggesting a high degree of relatedness. However, the CamBx3 strain exhibited lower similarity, with the lowest ANI and AAI values among the strains (Supplementary Figure 1; Figure 3). The lower similarity of CamBx3 may be related to its distinct ecological origin (hot spring habitat).

**Table 1:** Attributes of *Bacillus paralicheniformis* strains isolated from different ecological niches.

**Sr.no**.	**Name**	**Location**	**Isolation source**	**Size (Mbp)**	**CDS**	**GC%**	**Potential function**
1	*Bacillus paralicheniformis* 14DA11	South Korea	Doenjang (high-salt fermented soybean)	4.55	4.370	45.8	Antibiotic resistance
2	*Bacillus paralicheniformis* 285-3	China	Environmental	4.32	4.149	46	-
3	*Bacillus paralicheniformis* A4-3	South Korea	Tomato	4.58	4.487	45.76	-
4	*Bacillus paralicheniformis* ATCC9945a	-	-	4.38	4.217	45.9	Urease activity
5	*Bacillus paralicheniformis* Bac48	Saudi Arabia	Red sea lagoons -mangrove mud	4.46	4.201	45.9	Antimicrobial property
6	*Bacillus paralicheniformis* Bac84			4.38	4.237	45.8	
7	*Bacillus paralicheniformis* BL-09	China	Natural fermented sour congee	4.39	4.263	45.9	
8	*Bacillus paralicheniformis* CamBx3	-	Hot springs	4.45	4.371	45.8	-
9	*Bacillus paralicheniformis* CBMAI1303	Brazil	-	4.48	4,355	45.9	Biocontrol
10	*Bacillus paralicheniformis* CPL618	China, Jiangsu	Wheat field soil	4.45	-	45.9	Antimicrobial property
11	*Bacillus paralicheniformis* HAS-1	Mexico	Hypersaline soil	4.43	3,329	45.6	Antibacterial property
12	*Bacillus paralicheniformis* SUBG0010	India	Rhizosphere	4.32	4,167	45.9	
13	*Bacillus paralicheniformis* TXO7B-1SG6	Mexico	Hypersaline soil	4.52	4,159	45.9	
14	*Bacillus paralicheniformis* J25TS1	Japan	Honey	4.39	4,243	45.9	-
15	*Bacillus paralicheniformis* J36TS2			4.6	4,433	45.49	
16	*Bacillus paralicheniformis* J41TS8			4.5	4,356	45.8	
17	*Bacillus paralicheniformis* MDJK30	China	Rhizosphere	4.35	4,152	45.9	Plant Growth-Promoting and antimicrobial property
18	*Bacillus paralicheniformis* MHN12	India	Nodules	4.25	4,146	45.90	Potential use as bioinoculants
19	*Bacillus paralicheniformis* NCTC8721	-	-	4.43	4,221	45.8	-
20	*Bacillus paralicheniformis* RSC-1	Saudi Arabia	Sea water	4.32	4,122	46	Cellulase production
21	*Bacillus paralicheniformis* RSC-2			4.32	4,045	46	
22	*Bacillus paralicheniformis* RSC-3			4.32	4,674	46	
23	*Bacillus paralicheniformis* FA6	China, Hubei	Grass carp	4.45	4,289	45.9	Probiotic

### 3.3 Comparative pan-genome and secondary metabolite clusters analysis

**Figure 4:**
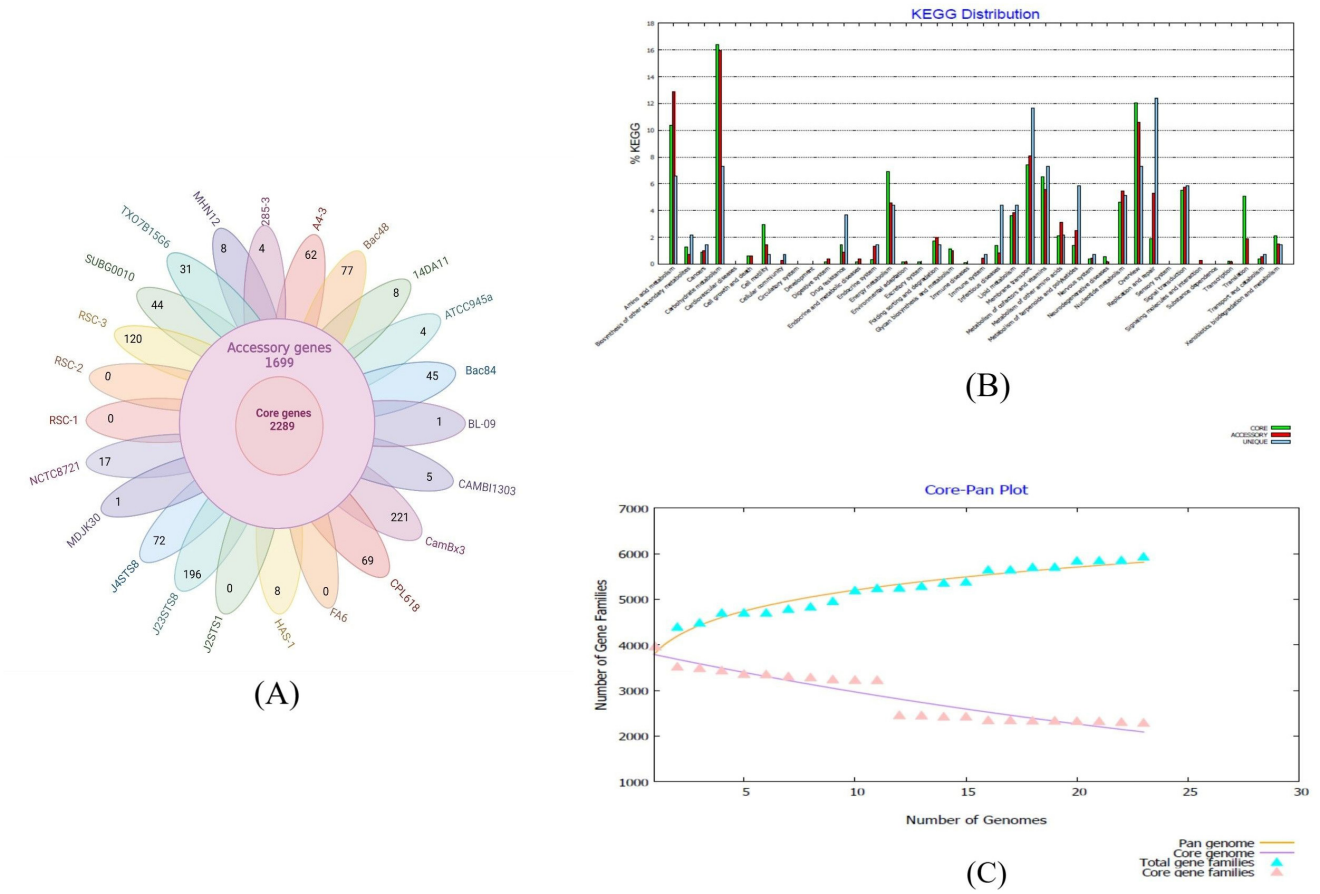
**(A)** Venn diagram depicting the distribution of core, accessory, and unique genes among the analyzed *Bacillus paralicheniformis* strains **(B)** Panel represents the functional distribution of genes based on Kyoto Encyclopedia of Genes and Genomes (KEGG) pathway annotation **(C)** Visual representation of the core-pan plot of *Bacillus paralicheniformis* strains.

A pan-genome analysis using the Bacterial Pan Genome Analysis Pipeline (BPGA) revealed 2,289 core and 1,699 accessory genes across the strains. Strains CamBx3 and J36TS2 had the highest numbers of unique genes i.e., 221 and 196 respectively. In contrast, strains RS1, RS2, J25TS1, and FA6 exhibited minimal unique genes (Figure 4A). Kyoto Encyclopedia of Genes and Genomes (KEGG) pathway analysis revealed that the core genome primarily encompassed genes involved in translation, cell motility, energy production, and carbohydrate metabolism. The accessory genome was enriched with pathways for amino acid metabolism including cysteine, methionine (*met*EGHB; *cys*E), arginine and proline (*arg*HEJBFID; *pro*AB), and biosynthesis of valine, isoleucine (*ilv*DGBIA), and tryptophan (*trp*ABCDEFSP). The unique genome prominently featured pathways for replication and repair (nucleotide excision, mismatch repair, homologous recombination), membrane transport, terpenoid, and polyketide metabolism (including fengycin synthesis and surfactant production), drug resistance (cationic antimicrobial peptide resistance), and secondary metabolite biosynthesis (Figure 4B). Pan-genome and core-genome analyses showed an expanding pan-genome with each new genome addition, indicating an “open” pan-genome (Figure 4C).

**Figure 5:**
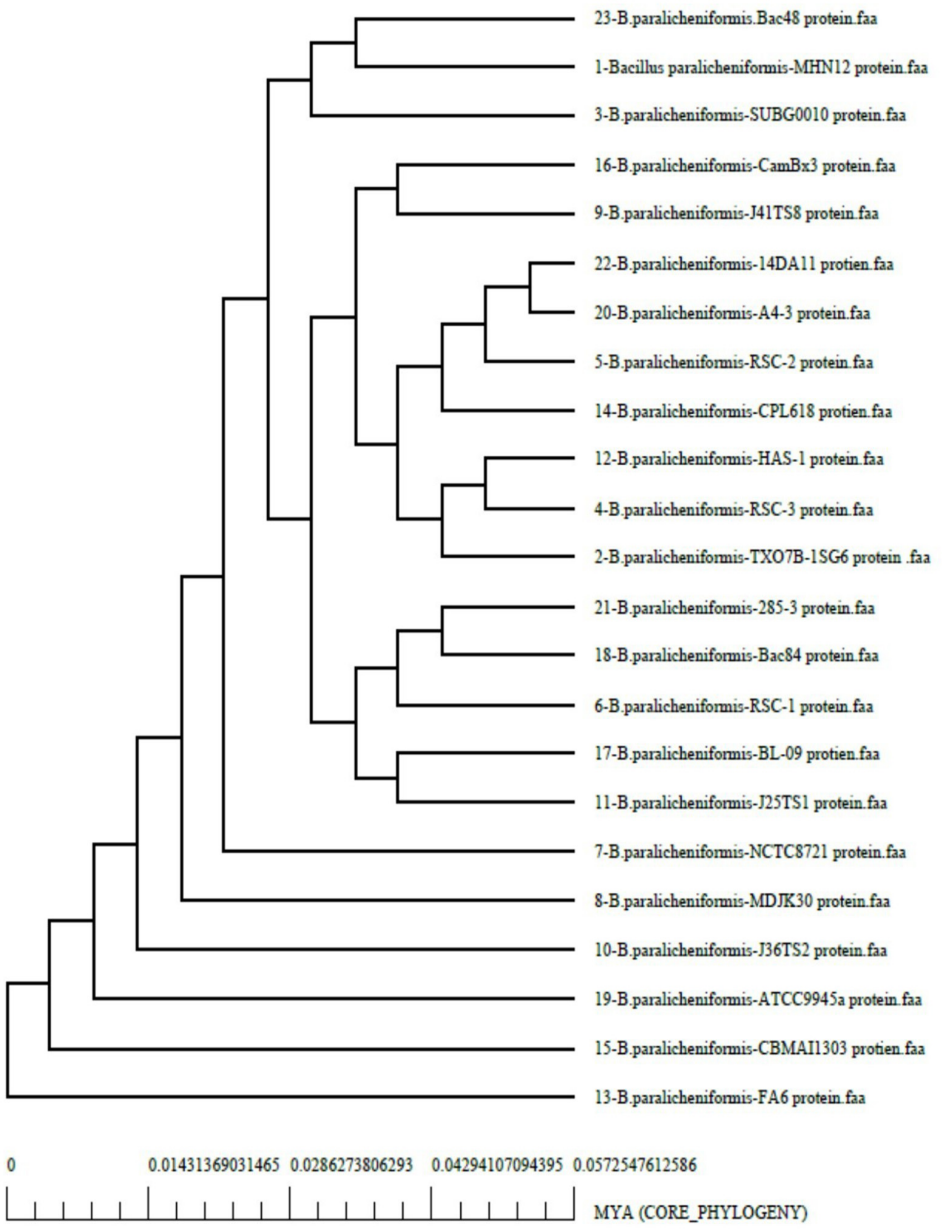
The phylogenetic relationships among *Bacillus paralicheniformis* strains based on the analysis of core genome.

**Figure 6:**
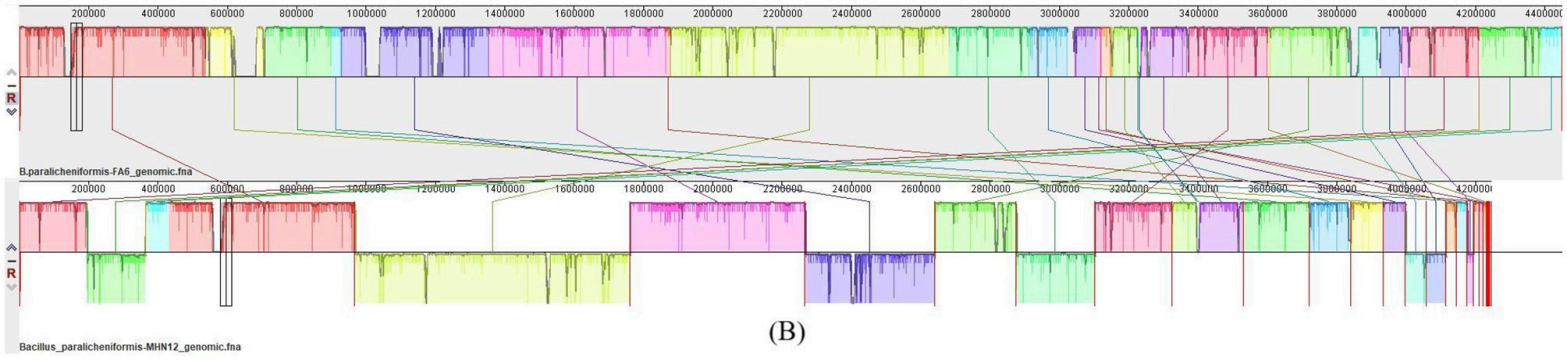
*Bacillus paralicheniformis* FA6 and *Bacillus paralicheniformis* MHN12 genomes compared using mauve.

Analyzing the core and pan-genomes revealed the evolutionary relationships between the strains. In the core genome phylogeny (Figure 5), strain MHN12 clustered closest to BAC48 and SUBG0010, all sharing a common trait of antimicrobial activity. The rest of the strains FA6, CBMAI1303, ATCC, J36TS2, MDJK, and NCTC formed distinct branches, each with distinct functional potentials. Notably, strain FA6, the most diverse among these, was recognized for its probiotic potential. In the pan-genome phylogeny (Supplementary Figure 3), strains BAC48, BAC84, RSC strains (1–3), 285-3, SUBG0010, and strain MHN12 clustered together, indicating a shared genetic lineage. The group possessed both antimicrobial and cellulase-producing capabilities. Genome comparison of different *B. paralicheniformis* strains was performed using BRIG v0.95. It revealed a high degree of similarity between strain MHN12 and BAC84, as indicated by the color intensity (Supplementary Figure 4). The genomes of strain FA6 and strain MHN12 were compared using Mauve 2.3.1 software (Figure 6). The genomic alignment identified 19 collinear blocks and revealed numerous inversion and rearrangement sites, indicating substantial genome shuffling between the strains. Overall, the genomes exhibited moderate conservation, with various sequences represented by the same-colored blocks. However, the presence of white spaces suggested insertions or deletions in specific regions, contributing to the observed differences between the two strains.

**Figure 7:**
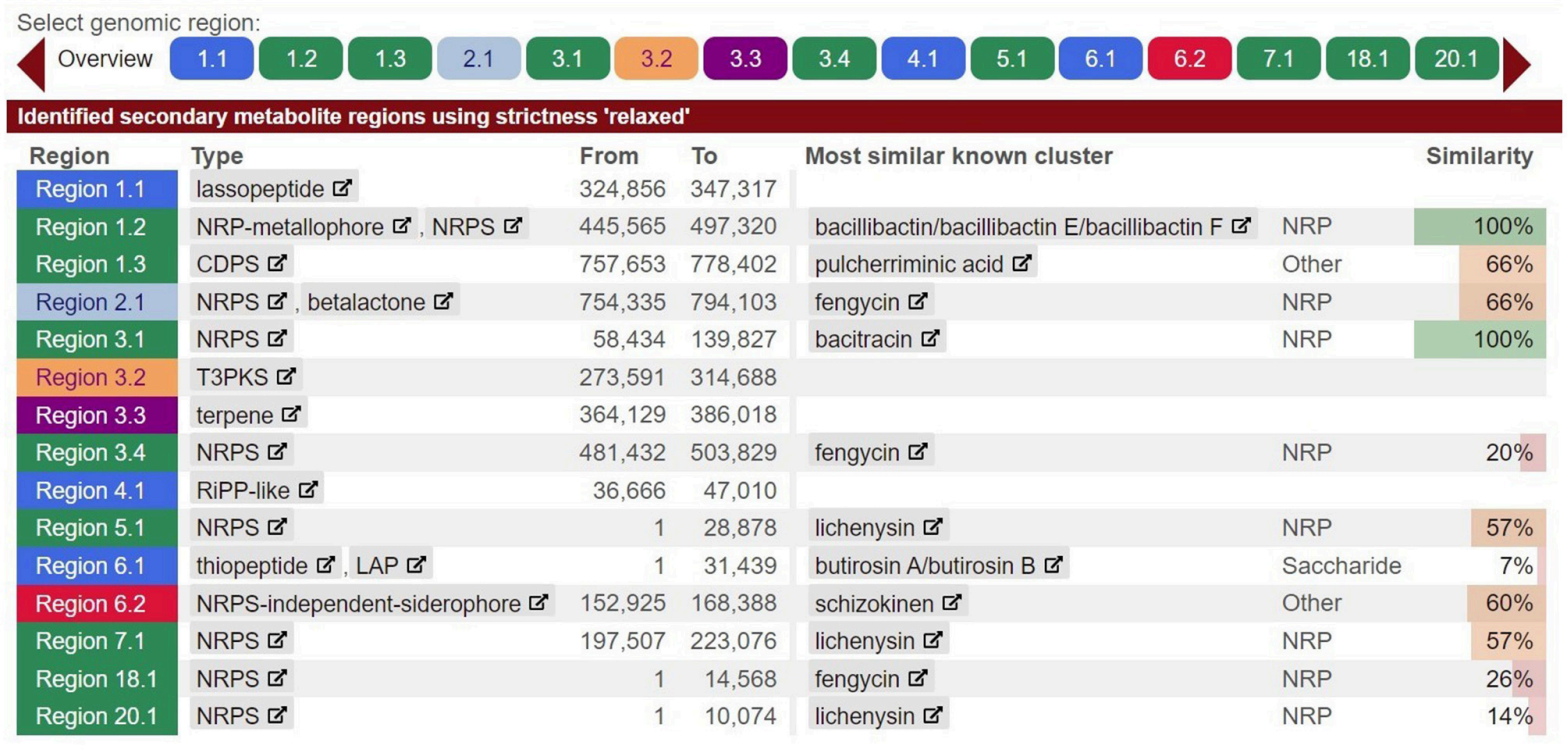
Graphical representation of predicted secondary metabolite cluster of *Bacillus paralicheniformis* MHN12.

The analysis of secondary metabolite clusters using antiSMASH software provided insights into the diverse biosynthetic capabilities of these strains. Each strain possessed 7 to 9 gene clusters, with strains CPL618 and HAS-1 displaying the most diverse secondary metabolite clusters. Most of the strains had complete similarity (100%) for gene clusters responsible for synthesizing lichenysin, bacillibactin, and bacitracin. However, strains BAC48, TX07B, and strain MHN12 showed variations in this pattern. BAC48 and TX07B displayed 88% similarity for the bacitracin operon, while strain MHN12 had only 57% similarity for the lichenysin operon (Figure 7). The gene clusters for fengycin synthesis were present in all the strains.

### 3.4 Pathway analysis and genomic insights into plant growth promotion abilities of *Bacillus paralicheniformis* MHN12

#### 3.4.1 Nutrient acquisition

The MinPathway analysis revealed several nutrient acquisition and utilization pathways within the strain MHN12 genome. The pathways included diverse functions such as ammonia assimilation, alkyl phosphonate utilization, ABC phosphate transport, alkanesulfonate utilization, iron-sulfur cluster assembly, and siderophore biosynthesis and transport. The strain MHN12 genome contained the *nas*ABCDE gene cluster for assimilatory nitrate reduction converting nitrate to ammonium. In addition, the genome had genes encoding extracellular nitrate/nitrite transporter (*nrk*), ammonium transporter (*nrg*AB, *amt*), anaerobic nitric oxide reductase (*nor*B), nitric oxide dioxygenase (*hmp)*, nitrate reductase (*nar*GHI), glutamine synthetase/glutamate synthase and glutamate dehydrogenase (*gln*A, *glt*BD). Furthermore, the presence of the ncd gene for nitronate monooxygenase highlight ability of strain MHN12 to metabolize nitroalkanes (Supplementary Table 1).

The strain harbored genes for producing organic acids such as gluconic acid, 2-keto gluconic acid, and others. Genes encoding phosphatases (*pho*ABD, *ppa*X), and phytase were annotated. Strain MHN12 also contained *pst*SABC genes for a high-affinity phosphate transport system. The annotation of gene clusters *dhb*ABCEF and *fhu*ABDG essential for siderophore synthesis and transport of ferric-siderophore complexes respectively supported the ability of strain MHN12 in enhancing iron availability. The *ssu*ABCDE gene cluster responsible for sulfonates assimilation to sulfite along with *cys*JI genes for sulfite reduction to sulfide (H_2_S) synthesis were identified in the strain MHN12 (Supplementary Table 1).

#### 3.4.2 Phytohormone and volatile compound production

The MinPath analysis highlighted key pathways, including tryptophan, acetoin, and 2,3-butanediol biosynthesis. Genes such as tryptophan 2-monooxygenase, amidase (*ami*E, *yaf* V), gibberellic acid (*car*AB), and tRNA prenylation for cytokinin biosynthesis (*mia*AB; *deo*) were identified. Strain MHN12 also possessed genes encoding α-acetolactate synthase, α-acetolactate decarboxylase, and butanediol dehydrogenase, key enzymes for volatile organic compound (VOC) synthesis, i.e., acetoin and 2,3-butanediol (Supplementary Table 1).

#### 3.4.3 Competitive and antagonistic behavior

The presence of the bacitracin stress response was revealed by the MinPathway analysis. The presence of *bce*ABRS, *bcr*ABC, and *spa*FKRGE operons essential for producing bacitracin, subtilin, and providing self-immunity were annotated. Genes responsible for polyamine production and transport (putrescine and spermidine) were identified (Supplementary Table 1).

#### 3.4.4 Motility, chemotaxis, and attachment to plant surface

The MinPathway analysis identified pathways motility, chemotaxis, exopolysaccharide production, flagella synthesis, and biofilm formation in the strain MHN12 genome. The identified key operons, including *fli*TRLCWDSQPNMJHGFE, *flg*MBLKGCDEF, *mot*AB, *sec*ADEFGY, and *flh*ABF are essential for constructing the flagellar apparatus. The genes associated with exopolysaccharide synthesis (*eps*ABDEFGNLM) were also found (Supplementary Table 1), supporting the strain's potential to form biofilms.

#### 3.4.5 Osmotic and oxidative stress tolerance

The key pathways for osmotic stress resilience included those for the uptake and biosynthesis of proline, choline, betaine, and trehalose. The genome harbored dedicated gene clusters for their biosynthesis (*pro*ABC, *glt*BD, *bet*ABC) and specific transporters (*pro*PXWV, *bet*L, *tre*P, *opu*ABCD) for efficient uptake. Additionally, genes essential for ion homeostasis, such as sodium/hydrogen antiporters (*nha*CP, *mrp*GFEDCAB) and potassium regulation systems (*kdp*ABDC, *kch, ktr*ABCD, *nha*P), further contribute to osmotic resilience of strain MHN12. The genome of strain MHN12 also featured antioxidant genes like superoxide dismutase, catalase, alkyl hydroperoxide reductase, glutathione peroxidase, as well as nitric oxide reductase (*nor*BQ), and flavohemoglobin (*hmp*) (Supplementary Table 1), all of which contribute to its capability to mitigate oxidative stress.

#### 3.4.6 Chaperones and spore formation

The MinPathway analysis revealed various genetic pathways essential for spore formation, including the Bsub Spore Coat, Sporulation Cluster IIIA, Spore Germination, Spore Coat, Small Acid-Soluble Spore Proteins, and Dipicolinate Synthesis. The presence of the Protein Chaperone pathway was evidenced by the annotation of genes encoding diverse chaperones like *hsp*33, *gro*EL, *gro*ES, *dna*KJ, *hrc*A, *grp*E, *clp*CPX, *htp*XG, and *csp*A (Supplementary Table 1).

#### 3.4.7 Heavy metal resistance

The MinPathway and genomic analysis revealed pathways and genes associated with resistance to heavy metals. The pathways included copper homeostasis, arsenic resistance, and manganese transport. The strain MHN12 genome featured genes for arsenate (*ars*CRB), zinc (*znu*ABCR), cadmium (*czr*A, *czc*D), copper (*cop*ZA, *ycn*JK), manganese, nickel (*nik*ABCDE), and chromate (*chr*) resistance. Genes related to magnesium-cobalt transport and homeostasis (*tly*C) were also identified (Supplementary Table 1).

### 3.5 *In vitro* PGP traits and stress specific metabolites under salinity stress

The isolate produced indole acetic acid (IAA) under control conditions (20 μg/mL), but no production was observed under stress. A slight reduction was noted in other PGP traits in stress conditions, including phosphate solubilization (from 890.94 to 802.23 μg/mL), ammonia production (from 2017.43 to 2000.25 μM/mL), and HCN synthesis. The production of stress-related metabolites was maintained or enhanced under stress. Exopolysaccharide (EPS) levels remained stable (from 1.003 to 0.97 mg/100 mL), while the activities of the antioxidant enzymes superoxide dismutase (SOD) and catalase (CAT) increased from 3.89 to 3.93 IU/mL and from 0.03 to 0.14 IU/mL, respectively.

### 3.6 Effect of salinity stress on the metabolite profile of MHN12

The major compounds under control conditions were cyclopropanedodecanoic acid (46.24%), pyrrolo[1,2-a] pyrazine-1,4-dione (57.43%), hexanoic acid (49.16%) and n-hexadecanoic acid (palmitic acid; 56.10%). Under salt stress, the relative abundance of n-hexadecanoic acid, pentanoic acid, and cyclopropanetetradecanoic acid decreased to 10.62%, 22.64%, and 27.05%, respectively. Furthermore, several compounds including tetradecanoic acid, dodecanoic acid, and octadecenoic acid were only noted in the control samples whereas oleic acid, hexadecanol, nonenoic acid were present only under salt stress. The concentration of pyrrolo [1,2-a] pyrazine-1,4-dione, hexahydro-3-(2-methylpropyl) increased with NaCl addition with probability % of 74.32% (Supplementary Table 2). Genes for bacterial fatty-acid biosynthesis (*fab*BDFGHILZ), along with a desaturase gene (*des*A) encoding an acyl-lipid desaturase enzyme involved in unsaturated fatty acid synthesis were also annotated in the strain MHN12 genome (Supplementary Table 1).

## 4 Discussion

Endophytes often outperform rhizospheric bacteria in promoting plant growth and stress resistance (Peng et al., 2023). The beneficial effects of these endophytes have been reported to be upregulated under abiotic stresses (Biswas et al., 2023). However, their implementation is hindered by a limited understanding of their action mechanisms (Peng et al., 2023; Zamanzadeh-Nasrabadi et al., 2023). *Bacillus paralicheniformis* strain MHN12 isolated from nodules of *Vigna radiata* has been screened for halotolerance, biocontrol property, plant-beneficial traits, and improved plant growth under lab conditions (Bhutani et al., 2021b, 2022). The current study addresses the gap in their implementation by focusing on genome sequencing, comparative analysis, and genome mining of strain MHN12.

Within the genus *Bacillus*, accurate differentiation between *B. licheniformis* and *B. paralicheniformis* remains a significant challenge due to their remarkable genetic similarity. 16S ribosomal RNA (rRNA) gene sequencing is insufficient for definitive identification, leading to frequent misclassifications (Olajide et al., 2021). This was also evident with strain MHN12 initially identified as *B. licheniformis* based on its 16S rRNA sequence. However, recent comparative genomic studies revealed the presence of specific secondary metabolite operons (fengycin, paralichenicidin, and bacitracin) exclusively in *B. paralicheniformis* genomes, absent in *B. licheniformis*. This discovery prompted us to do the whole genome sequencing of the isolate to analyze its secondary metabolite cluster genes, and perform its comparative analysis with the other reported *B. paralicheniformis* strains to ascertain its identity and draw the similarities and differences among them. The dDDH analysis with a 70% similarity threshold for genome boundaries and ANI and AAI analysis using a 95–96% cut-off delimiting species confirmed that all the strains belong to the same genus and species (Aliyu et al., 2016). The pan-genome analysis is a widely used approach among the currently available comparative genomic methods to evaluate the genomic diversity of entire repertoires. It helps identify core genomes (shared by all strains), accessory genomes (found in two or more strains), and specific gene pools unique to individual strains within a species (Tettelin et al., 2008). The core genome of the strains included genes responsible for the basic functioning. The accessory and unique genome encoded genes for biochemical pathways and functions that confer selective advantages, such as antibiotic resistance, drug resistance, and secondary metabolite biosynthesis. The pan-genome phylogeny revealed that strain MHN12 grouped with strains possessing accessory genes for antimicrobial and cellulase production, suggesting the acquisition of traits important for plant–microbe interactions and competitive fitness. This is further supported by the core–pan plot analysis, which indicated an ‘open' pan-genome, reflecting high genomic flexibility and a strong potential for acquiring new genes through horizontal gene transfer (Bach et al., 2022). Such genomic openness may explain how strain MHN12 and related strains gain beneficial functions such as improved environmental resilience, probiotic potential. The antiSMASH analysis identified characteristic secondary metabolite clusters (lichenysin, bacillibactin, bacitracin, and fengycin) in the genome of strain MHN12 and other strains. This finding confirmed their taxonomic classification as *B. paralicheniformis*, since the presence of bacitracin and fengycin biosynthetic clusters have been reported in *B*. *paralicheniformis* only when compared to *B. licheniformis*. Secondary metabolites synthesized by plant endophytes can improve plant resilience against different biotic and abiotic stresses (Wang et al., 2022). Secondary metabolite gene clusters lichenysin, bacillibactin, bacitracin, and fengycin, with roles in biosurfactants, iron uptake, antibacterial, and antifungal activities respectively, contribute to inhibiting pathogens and improving nutrient uptake were predicted in the genome (Harwood et al., 2018). In support of this potential, strain MHN12 demonstrated biocontrol activity against *Fusarium oxysporum* and *Aspergillus niger* (Bhutani et al., 2021b). The finding resonates with a genome mining study of *B. paralicheniformis* ES-1, which reported similar gene clusters associated with *in vitro* antibacterial activity and overall plant health improvement (Iqbal et al., 2023). Moreover, the GC-MS analysis revealed the presence of compounds such as piperidinone, 2,4-di-tert-butylphenol, and 3-trifluoroacetoxydodecane, with known antimicrobial properties (Das et al., 2025).

**Figure 8:**
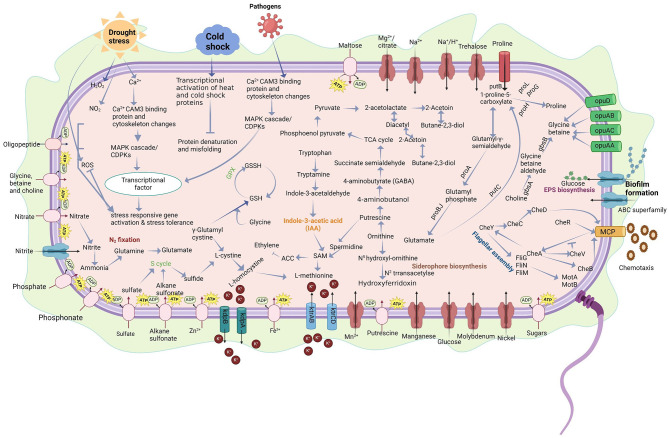
An overview of the reconstructed metabolic pathways of *Bacillus paralicheniformis* MHN12 based on KEGG annotations, highlighting key pathways associated with plant growth promotion and stress tolerance.

In line with the plant growth-promoting (PGP) traits and salt stress resilience of strain MHN12, various pathways and genes linked to stress resistance, phosphate solubilization, nitrogen metabolism and transport, phytohormone production, siderophore, acetoin, and butanediol synthesis were identified in its genome (Figure 8). Strain MHN12 genome illustrated the presence of genes for N, P, and Fe acquisition and transporters for better uptake of soil nutrients. Plant growth-promoting endophytic bacteria (PGPEBs) convert non-usable forms of nitrogen into forms usable by plants, such as ammonium. The presence of genes for nitrate and urea conversion into ammonia further validates the findings of Bhutani et al. (2021a),b on ammonia production by strain MHN12 (Bhutani et al., 2021b). Additionally, the presence of an ammonium assimilation enzyme system regulates nitrogen absorption and transformation, thereby providing nitrogen for plant growth. Polyamines such as putrescine, whose biosynthetic genes were annotated in strain MHN12, plays role in enhancing plant performance under nitrogen limitation. The transgenic *Arabidopsis* overexpressing gene for putrescine biosynthesis support the role of polyamines in improving plant performance under nitrogen limitation. The elevated putrescine levels were associated with enhanced nitrogen metabolism and strengthened antioxidant defenses by increased nitrate uptake and nitrate reductase activity as well as reducing oxidative damage (Recalde et al., 2025). Altogether, the data obtained depicts that strain MHN12 may modulate plant nitrogen levels directly. Additionally, the presence of genes for nitric oxide reductase and nitric oxide dioxygenase suggests a potential role for strain MHN12 in modulating nitric oxide (NO) signaling. NO serves as a crucial signaling molecule in plant growth and plant-microbe interactions (Mur et al., 2013).

Phosphate solubilization is a crucial process as phosphorus has limited bioavailability in many environments due to its complex formation with metal ions. Phosphate-solubilizing bacteria effectively convert insoluble phosphorus into plant-available forms by producing acid and alkaline phosphatases (Swain et al., 2012). Previous studies have shown that alkaline phosphatase is predominantly produced by soil microorganisms. The prokaryotic genes *pho*XAD encode alkaline phosphatases, with *pho*D acting as potential biomarker for assessing phosphate solubilization (Wan et al., 2020). The strain MHN12 genome harbored genes for alkaline phosphatase (*pho*ABD), pyrophosphatase (*ppa*X), and phosphonate degradation and transport systems (*pst*ABCS). Similar gene clusters have also been reported in plant growth-promoting phosphate solubilizing *B. velezensis* strains (Ercole et al., 2024).

Iron is a co-factor in many processes and activities including photosynthesis and oxidative phosphorylation thus essential for plant growth. Siderophores produced by endophytes can aid plants growing in an iron-deficient environment. Siderophores form complexes with iron to improve iron availability in plants' rhizosphere and meet plant growth's nutritional needs (Garzón-Posse et al., 2019). The strain MHN12 produced siderophores and possessed operon (*dhb*) involved in synthesizing bacillibactin siderophore (Bhutani et al., 2021b). Similar gene clusters for different nutrient acquisition were annotated in other plant growth-promoting strains like *B. paralicheniformis* RP01 and *B. licheniformis* TAB7 (Mpofu et al., 2019; Xu et al., 2023). Bacteria usually code for more siderophore uptake systems that enable them to efficiently sequester siderophores produced by other microorganisms (xenosiderophores). For example, *Escherichia coli* can assimilate ferrichrome and ferrioxamine siderophores through the *fhu*BCD transport system (Llamas et al., 2006; Hannauer et al., 2010; Miethke et al., 2013), and similar operons were identified in the strain MHN12 genome. Sulfur assimilation in bacteria can occur through the uptake of organic molecules, including sulfate esters, and sulfonates. The strain MHN12 exhibited a gene cluster for sulfur assimilation from sulfonates, an operon identified across diverse bacterial taxa including *B. subtilis, Escherichia coli*, and *Pseudomonas putida* (Coppée et al., 2001; Koch et al., 2005). This suggests a conserved sulfonate utilization strategy among various bacteria.

Phytohormones such as indole acetic acid (IAA), gibberellins, and cytokinins play a direct role in enhancing crop productivity (Orozco-Mosqueda et al., 2023). The strain MHN12 produced IAA in the presence of exogenous tryptophan (Bhutani et al., 2021b) and its genome analysis also revealed the presence of the genes for tryptophan and IAA biosynthesis via the indole-3-acetamide (IAM) pathway. IAA production is a common feature in around 70% of assessed halotolerant PGPB with IAM and indole-3-pyruvic acid (IPyA) pathway genes found in 60% and 80% of these bacteria, respectively (Zamanzadeh-Nasrabadi et al., 2023). Volatile organic compounds such as acetoin and 2,3-butanediol whose synthesis operons were annotated in the strain MHN12 genome play significant roles in stimulating plant growth, inducing systemic resistance, enhancing drought resilience, and influencing phytohormone profiles (Orozco-Mosqueda et al., 2023). *Arabidopsis thaliana* plantlets exposed to acetoin and 2,3-butanediol produced by *B. amyloliquefaciens* showed antagonistic activity and better growth (Wu et al., 2019).

Polyamines like putrescine and spermidine play essential roles in bacterial stress resilience and plant-microbe interactions. Under stress, plant ethylene levels increase significantly, negatively impacting growth and promoting senescence. The presence of multiple polyamine biosynthesis and transport genes in the strain MHN12 genome suggests its ability to control plant S-adenosyl methionine (SAM) levels, a precursor for ethylene, and influence ethylene biosynthesis enzymes (ACC synthase and ACC oxidase) via spermidine production. Additionally, strain MHN12 has been screened positive for ACC deaminase activity, an enzyme that degrades the immediate ethylene precursor, 1-aminocyclopropane-1-carboxylic acid (ACC), promoting plant growth (Xie et al., 2014; Maheshwari et al., 2020; Bhutani et al., 2021b). These findings suggest that strain MHN12 utilizes a dual strategy: modulating SAM and directly degrading ACC, to regulate plant ethylene levels and consequently promote plant growth.

The beneficial plant-promoting effects of bacteria primarily lie in the successful colonization of host plants by endophytes. Bacterial colonization in plants begins with the recognition of chemical signals released by roots, followed by chemotactic responses to these signals. Bacteria then migrate toward the roots and colonize different plant parts (Kumar et al., 2020). Genomic analysis revealed genes related to chemotaxis, and flagella synthesis suggesting strong chemotactic response and motility abilities of strain MHN12. Genes involved in chemotaxis and motility are important for the colonization abilities of plant-associated bacteria, particularly endophytes (Hardoim et al., 2015). Several genes involved in exopolysaccharide synthesis were identified in the strain MHN12 genome. Exopolysaccharide (EPS) has been reported to contribute to plant salt tolerance by chelating free Na^+^ from the soil, restricting Na^+^ entry into plants, facilitating biofilm formation, and improving soil stability (Liu et al., 2022). The addition of EPS from *Pseudomonas simiae* MHR6 effectively improved Na^+^/K^+^ homeostasis in maize under salt stress (Liu et al., 2023).

The *in vitro* analysis of PGP traits as well as production of stress aiding metabolites under salinity revealed that the isolate was able to maintain its PGP traits i.e., phosphate solubilization, HCN and ammonia production even under stress. Moreover, enhanced antioxidant activity and increased EPS production may help strain MHN12 in maintaining ionic balance and thereby support its ability to cope with salinity stress. Additionally, the presence of genes for chaperones, antioxidant enzymes, and heavy metal resistance, further aids strain MHN12 to effectively combat various abiotic stresses, including thermal, oxidative, and heavy metal stress. The strain MHN12 genome harbored genes for osmolyte production and transporters crucial for cell turgor maintenance under salinity or osmotic stress. These osmolyte transporters facilitate cellular osmotic adjustments (Parmar et al., 2017). Experimentally, strain MHN12 has demonstrated the ability to tolerate and grow in high salinity, up to 15% NaCl (2.6 M) and it can produce increased amounts of proline under stress conditions, an important osmolyte (Bhutani et al., 2022).

The plasma membrane functions as the primary barrier protecting bacteria from environmental stresses. Under saline conditions, membrane function is disrupted, causing altered permeability, loss of fluidity, and impaired selective ion transport. A key adaptive response to this challenge is the regulation of membrane fluidity through the modulation of fatty acid composition (Wang et al., 2020; Rojas-Solis et al., 2020). The annotation of desaturase gene (*des*A) supports strain MHN12 capacity for unsaturated fatty-acid synthesis. Moreover, a decrease in saturated fatty acids (SFAs) including palmitic acid (n-hexadecanoic acid), stearic acid (octadecanoic acid), and myristic acid (tetradecanoic acid) and an increase in unsaturated fatty acids (UFAs) such as oleic acid was observed. Similarly, in other osmotolerant organisms such as *Bacillus* sp. CR71, *Zygosaccharomyces rouxii, Tetragenococcus halophilus*, and *Debaryomyces hansenii*, a decrease in the saturated to unsaturated fatty acid (SFA/UFA) ratio under salt stress has been observed contributing to maintain optimal membrane fluidity (He et al., 2017; Wang et al., 2020; Rojas-Solis et al., 2020). An increase in fatty acid unsaturation in membrane lipids enhances membrane fluidity, which supports the proper functioning of membrane-embedded ion transport systems (such as Na^+^/H^+^ antiporters), thereby helping maintain ion homeostasis under salt stress (Allakhverdiev et al., 2001). The significant increase in pyrrolo [1,2-a] pyrazine-1,4-dione, hexahydro-3-(2-methylpropyl) under salt stress highlights its protective role. It is a non-enzymatic antioxidant capable of mitigating oxidative damage induced by stress conditions (Nas et al., 2021). This resilience and genomic potential of strain MHN12 support it to adapt to salinity. Future studies focusing on *in planta* assays are essential to validate efficacy of strain MHN12 in alleviating salt stress and promoting plant growth under field conditions.

## 5 Conclusion

The genetic proximity between *Bacillus paralicheniformis* and *Bacillus licheniformis* often leads to misclassification of the former, as evidenced by the initial identification of strain MHN12 as *B. licheniformis* in the present work. However, the presence of secondary metabolite operons specific to *B. paralicheniformis* and absent in *B. licheniformis* distinguishes strain MHN12 from *B. licheniformis*. Detailed genomic analysis of strain MHN12 revealed several pathways and a multitude of genes facilitating plant colonization, growth promotion, and stress tolerance. The genome annotation confirmed the presence of genes responsible for key plant growth-promoting and stress resilience functions, such as indole-3-acetic acid (IAA) and siderophore production, 1-aminocyclopropane-1-carboxylate (ACC) deaminase activity, phosphate solubilization using alkaline phosphatase, proline, and antioxidant enzyme synthesis. The presence of genes for acetoin, 2,3-butanediol, and secondary metabolite biosynthesis indicated the strain's potential for bio-control and defense. *In vitro* assays validated the genomic data, confirming ability of strain MHN12 to exhibit plant growth promoting traits along with production of enzymatic antioxidants and exopolysaccharides (EPS) under salinity stress. Additionally, the observed decrease in the SFA/UFA ratio under salt stress represented a vital adaptive strategy of strain MHN12 toward harsh conditions. This analysis underlines the adaptability of *B. paralicheniformis* MHN12 in enhancing plant growth, even in saline environments. Further research is necessary to elucidate the precise functions and regulatory mechanisms of these genes. The large-scale green house as well as field trials are essential to validate the strain's performance under diverse agro-climatic and saline conditions.

## Data Availability

The datasets presented in this study can be found in online repositories. The names of the repository/repositories and accession number(s) can be found in the article/Supplementary material.
