# Supplementary material for: Integrative genomic and functional characterization of halotolerant Bacillus paralicheniformis MHN12 for sustainable agriculture
Source: Front Microbiol. 2026 Jan 23;16:1736288. doi: 10.3389/fmicb.2025.1736288 (PMC12876140; doi:10.3389/fmicb.2025.1736288)
Supplement: Supplementary file 1 [file Data_Sheet_1.docx]

Title: Integrative Genomic and Functional Characterization of Halotolerant *Bacillus paralicheniformis* MHN12 for Sustainable Agriculture

Priyanka Dahiya^1^, Shruti Dhiman^1^, Pradeep Kumar^1^, Simran Rani^1^, A Sankara narayanan^2^, Kiran Arora^3^, Amita Suneja Dang^4^, Pooja Suneja^1*^

Affiliations:

1 Plant-Microbe Interaction Laboratory, Department of Microbiology, Maharshi Dayanand University, Rohtak, Haryana, India

2 Department of Life Sciences, Sri Sathya Sai University for Human Excellence, Navanihal, Kalaburagi, Karnataka, India

3 Kirori Mal College, University of Delhi, Delhi-10007, India

4 Centre for Medical Biotechnology, Maharshi Dayanand University, Rohtak, Haryana, India

*Address correspondence to Pooja Suneja, [poojapavit@gmail.com](mailto:poojapavit@gmail.com)

Present address- Plant-Microbe Interaction Laboratory, Department of Microbiology, Maharshi Dayanand University, Rohtak 124001, Haryana, India

ORCID ID: 0000-0003-2697-9168

**Table S1.** Genes linked to plant growth promotion and stress resilience potential of MHN12.

| **Plant growth promoting and stress resilience activities** | **Gene name** | **Product** |
| --- | --- | --- |
| **Nutrient Acquisition** | | |
| **Nitrogen and ammonia metabolism** | *nas*A | Assimilatory nitrate reductase catalytic subunit |
|  | *nas*B | Nitrite reductase [NAD(P)H] large subunit |
|  | *nas*C | Assimilatory nitrate reductase catalytic subunit |
|  | *nas*D | Nitrite reductase [NAD(P)H] large subunit |
|  | *nas*E | Nitrite reductase [NAD(P)H] small subunit |
|  | *nrg*A | Ammonium transporter |
|  | *nrg*B | Nitrogen regulatory PII-like protein |
|  | *amt* | Ammonium Transporter, Amt family |
|  | *nor*B | Nitric oxide reductase subunit B |
|  | *hmp* | Nitric oxide dioxygenase |
|  | *nar*G | Nitrate reductase / nitrite oxidoreductase, alpha subunit |
|  | *nar*H | Nitrate reductase / nitrite oxidoreductase, beta subunit |
|  | *nar*I | Nitrate reductase gamma subunit |
|  | *ncd* | Nitronate monooxygenase |
|  | *ure*A | Urease subunit gamma |
|  | *ure*B | Urease subunit beta |
|  | *ure*C | Urease subunit alpha |
|  | *ure*E | Urease accessory protein UreE |
|  | *ure*F | Urease accessory protein UreF |
|  | *ure*G | Urease accessory protein UreG |
|  | *ure*D | Urease accessory protein UreD |
|  | *gln*A | Glutamine synthetase |
|  | *glt*B | Glutamate synthase (NADPH) large chain |
|  | *glt*D | Glutamate synthase (NADPH) small chain |
| **Polyamine biosynthesis** | *spe*A | Arginine decarboxylase |
|  | *spe*B | Agmatinase |
|  | *spe*D | S-adenosylmethionine decarboxylase |
|  | *spe*E | Spermidine synthase |
|  | *spe*G | Diamine N-acetyltransferas |
|  | ABC.SP.P | Putative spermidine/putrescine transport system permease protein |
|  | *puu*P | Putrescine importer |
| **Phosphate uptake and metabolism** | | |
|  | *pho*A | Alkaline phosphatase |
|  | *pho*B | Alkaline phosphatase |
|  | *pho*D | Alkaline phosphatase D |
|  | *ppa*X | Pyrophosphatase PpaX |
|  | *pst*S | Phosphate transport system substrate-binding protein |
|  | *pst*A | Phosphate transport system permease protein |
|  | *pst*B | Phosphate transport system ATP- binding protein |
|  | *pst*C | Phosphate transport system permease protein |

| **Iron acquisition** | | |
| --- | --- | --- |
|  | *dhb*A | 2,3-dihydro-2,3-dihydroxybenzoate dehydrogenase |
|  | *dhb*B | Isochorismatase |
|  | *dhb*C | Isochorismate synthase DhbC |
|  | *dhb*E | 2,3-dihydroxybenzoate-AMP ligase |
|  | *dhb*F | Dimodular nonribosomal peptide synthase |
|  | *fhu*A | Hydroxamate/heme transport system ATP-binding protein |
|  | *fhu*B | Hydroxamate/heme transport system permease protein |
|  | *fhu*D | Hydroxamate/heme transport system substrate-binding protein |
|  | *fhu*G | Hydroxamate/heme transport system permease protein |
| **Sulfonate assimilation** | | |
|  | *ssu*A | Sulfonate transport system Substrate- binding protein |
|  | *ssu*B | Sulfonate transport system ATP- binding protein |
|  | *ssu*C | Sulfonate transport system permease protein |
|  | *ssu*D | Alkanesulfonate monooxygenase |
|  | *ssu*E | Alkanesulfonate oxidoreductase |
| **Phytohormone synthesis** | | |
|  | *ami*E | Amidase |
|  | *yaf*V | Tryptophan 2-monooxygenase |
|  | *car*A | Carbamoyl-phosphate synthase small subunit |
|  | *car*B | Carbamoyl-phosphate synthase large subunit |
|  | *mia*A | tRNA dimethylallyltransferase |
|  | *mia*B | tRNA-2-methylthio-N6-  dimethylallyladenosine synthase |
| **Volatile Compound synthesis** |  |  |
|  | *ilv*K | Branched-chain-amino-acid aminotransferase 2 |
|  | *ilv*D | Dihydroxy-acid dehydratase |
|  | *ilv*A | L-threonine dehydratase biosynthetic IlvA |
|  | *Ilv*C | Ketol-acid reductoisomerase (NADP(+)) |
|  | *ilv*H | Acetolactate synthase small subunit |
|  | *ilv*B | Acetolactate synthase large subunit |
|  | *ilv*E | Branched-chain-amino-acid aminotransferase |
|  | *bud*A, | Acetolactate decarboxylase |
|  | *bud*C | Meso-butanediol dehydrogenase |
| **Bacitracin and subtilin production** | | |
| **Bacitracin production** | *bce*A | Bacitracin transport system ATP- binding protein |

|  | *bce*B | Bacitracin transport system permease protein |
| --- | --- | --- |
|  | *bce*R | Two-component system, OmpR  family, bacitracin resistance response regulator BceR |
|  | *bce*S | Two-component system, OmpR family, bacitracin resistance sensor histidine kinase BceS |
|  | *bcr*A | Bacitracin transport system ATP- binding protein |
|  | *bcr*B | Bacitracin transport system permease protein |
|  | *bcr*C | Undecaprenyl-diphosphatase |
| **Subtilin production** | *spa*F | Lantibiotic transport system ATP- binding protein |
|  | *spa*K | Two-component system, OmpR family, lantibiotic biosynthesis  sensor histidine kinase NisK/SpaK |
|  | *spa*R | Two-component system, OmpR family, lantibiotic biosynthesis response regulator NisR/SpaR |
|  | *spa*G | Lantibiotic transport system permease protein |
|  | *spa*E | Lantibiotic transport system permease protein |
| **Motility and Chemotaxis** | *fli*T | Flagellar protein FliT |
|  | *fli*R | Flagellar biosynthesis protein FliR |
|  | *fli*L | Flagellar protein FliL |
|  | *fli*C | Flagellin |
|  | *fli*W | Flagellar assembly factor FliW |
|  | *fli*D | Flagellar hook-associated protein 2 |
|  | *fli*S | Flagellar secretion chaperone FliS |
|  | *fli*Q | Flagellar biosynthesis protein FliQ |
|  | *fli*P | Flagellar biosynthesis protein FliP |
|  | *fli*N | Flagellar motor switch protein FliN |
|  | *fli*M | Flagellar motor switch protein FliM |
|  | *fli*J | Flagellar protein FliJ |
|  | *fli*H | Flagellar assembly protein FliH |
|  | *fli*G | Flagellar motor switch protein FliG |
|  | *fli*F | Flagellar M-ring protein FliF |
|  | *fli*E | Flagellar hook-basal body complex protein FliE |
|  | *flg*M | Negative regulator of flagellin synthesis FlgM |
|  | *flg*B | Flagellar basal-body rod protein FlgB |
|  | *flg*L | Flagellar hook-associated protein 3 FlgL |
|  | *flg*K | Flagellar hook-associated protein 1 |
|  | *flg*G | Flagellar basal-body rod protein FlgG |
|  | *flg*C | Flagellar basal-body rod protein FlgC |

|  | *flg*D | Flagellar basal-body rod modification protein FlgD |
| --- | --- | --- |
|  | *flg*E | Flagellar hook protein FlgE |
|  | *flg*F | Flagellar basal-body rod protein FlgF |
|  | *mot*A | Chemotaxis protein MotA |
|  | *mot*B | Chemotaxis protein MotB |
|  | *sec*A | Preprotein translocase subunit SecA |
|  | *sec*D | Fusion protein |
|  | *sec*E | Preprotein translocase subunit SecE |
|  | *sec*F | Fusion protein |
|  | *sec*G | Preprotein translocase subunit SecG |
|  | *sec*Y | Preprotein translocase subunit SecY |
|  | *flh*A | Flagellar biosynthesis protein FlhA |
|  | *flh*B | Flagellar biosynthesis protein FlhB |
|  | *flh*F | Flagellar biosynthesis protein FlhF |
| **Exopolysaccharide synthesis** | | |
|  | *eps*A | Protein tyrosine kinase modulator |
|  | *eps*B | Protein-tyrosine kinase |
|  | *eps*D | Glycosyltransferase EpsD |
|  | *eps*E | Glycosyltransferase EpsE |
|  | *eps*F | Glycosyltransferase EpsF |
|  | *eps*G | Transmembrane protein EpsG |
|  | *eps*N | Pyridoxal phosphate-dependent aminotransferase EpsN |
|  | *eps*L | Sugar transferase EpsL |
|  | *eps*M | Acetyltransferase EpsM |
| **Osmotic stress tolerance** | | |
|  | *pro*A | Glutamate-5-semialdehyde dehydrogenase |
|  | *pro*B | Glutamate 5-kinase |
|  | *pro*C | Pyrroline-5-carboxylate reductase |
|  | *glt*B | Glutamate synthase (NADPH) large chain |
|  | *glt*D | Glutamate synthase (NADPH) small chain |
|  | *gbs*A | Betaine-aldehyde dehydrogenase |
|  | *gbs*B | Choline dehydrogenase |
|  | *gbs*R | HTH-type transcriptional regulator, glycine betaine synthesis regulator |
|  | *put*P | Sodium/proline symporter |
|  | *pro*X | Glycine betaine/proline transport system substrate-binding protein |
|  | *pro*W | Glycine betaine/proline transport system permease protein |
|  | *pro*V | Glycine betaine/proline transport system ATP-binding protein |
|  | *bet*L | Glycine betaine transporter |
|  | *opu*AC | Glycine betaine-binding protein OpuAC |
|  | *opu*AB | Glycine betaine transport system permease protein OpuAB |
|  | *opu*AA | Glycine betaine transport ATP- binding protein OpuAA |

|  | *opu*D | Glycine betaine transporter OpuD |
| --- | --- | --- |
|  | *opu*CA | Glycine betaine/carnitine/choline transport ATP-binding protein OpuCA |
|  | *opu*CB | Glycine betaine/carnitine/choline transport system permease protein  OpuCB |
|  | *opu*CC | Glycine betaine/carnitine/choline- binding protein OpuCC |
|  | *opu*CD | Glycine betaine/carnitine/choline transport system permease protein OpuCD |
|  | *opu*E | Osmoregulated proline transporter OpuE |
|  | *nha*C | Na(+)/H(+) antiporter NhaC |
|  | *nha*P | K(+)/H(+) antiporter NhaP2 |
|  | *mrp*G | Na(+)/H(+) antiporter subunit G |
|  | *mrp*F | Na(+)/H(+) antiporter subunit F |
|  | *mrp*E | Na(+)/H(+) antiporter subunit E |
|  | *mrp*D | Na(+)/H(+) antiporter subunit D |
|  | *mrp*C | Na(+)/H(+) antiporter subunit C |
|  | *mrp*A | Na(+)/H(+) antiporter subunit A |
|  | *mrp*B | Na(+)/H(+) antiporter subunit B |
|  | *kdp*A | Potassium-transporting ATPase potassium-binding subunit |
|  | *kdp*B | Potassium-transporting ATPase ATP- binding subunit |
|  | *kdp*D | Sensor protein KdpD |
|  | *kdp*C | Potassium-transporting ATPase KdpC subunit |
|  | *kch* | Voltage-gated potassium channel |
|  | *ktr*A | System potassium uptake protein |
|  | *ktr*B | System potassium uptake protein |
|  | *ktr*C | System potassium uptake protein |
|  | *ktr*D | System potassium uptake protein |
|  | *nha*P | Potassium/hydrogen antiporter |
| **Oxidative stress tolerance** | | |
|  | *sod* | Superoxide dismutase, Fe-Mn family |
|  | *kat*A | Catalase |
|  | *kat*E | Catalase |
|  | *gpx* | Glutathione peroxidase |
|  | *ahp*C | Alkyl hydroperoxide reductase C |
|  | *tpx* | Thiol peroxidase |
|  | *nor*B | Nitric oxide reductase subunit |
|  | *nor*Q | Nitric oxide reductase subunit |
|  | *Hmp* | Nitric oxide dioxygenase |
| **Chaperones formation** | | |
|  | *gro*EL | Chaperonin GroEL |
|  | *gro*ES | Chaperonin GroES |
|  | *dna*K | Molecular chaperone DnaK |
|  | *dna*J | Molecular chaperone DnaJ |

|  | *hrc*A | Heat-inducible transcriptional repressor |  |
| --- | --- | --- | --- |
|  | *grp*E | Molecular chaperone GrpE |  |
|  | *clp*C | ATP-dependent Clp protease ATP- binding subunit clpC |  |
|  | *clp*P | ATP-dependent Clp protease, protease subunit |  |
|  | *clp*X | ATP-dependent Clp protease ATP- binding subunit ClpX |  |
|  | *htp*X | Heat shock protein HtpX |  |
|  | *htp*G | Molecular chaperone HtpG |  |
|  | *csp*A | Cold shock protein |  |
|  | *hsl*O | Molecular chaperone Hsp33 |  |
| **Heavy metal resistance** | | |  |
| **Arsenic resistance** | *ars*C | Arsenate reductase (thioredoxin) |  |
|  | *ars*R | ArsR family transcriptional regulator,  arsenate/arsenite/antimonite-  responsive transcriptional repressor |  |
|  | *ars*B | Arsenical pump membrane protein |  |
| **Zinc resistance** | *znu*A | Zinc transport system substrate- binding protein |  |
|  | *znu*B | Zinc transport system permease protein |  |
|  | *znu*C | Zinc transport system ATP-binding protein |  |
|  | *znu*R | Fur family transcriptional regulator, zinc uptake regulator |  |
| **Cadmium resistance** | *czr*A | ArsR family transcriptional  regulator, zinc-responsive transcriptional repressor |  |
|  | *czc*D | Cobalt-zinc-cadmium efflux system protein |  |
| **Copper resistance** | *cop*Z | Copper chaperone |  |
|  | *cop*A | P-type Cu+ transporter |  |
|  | *ycn*J | Copper transport protein |  |
|  | *Ycn*K | DeoR family transcriptional regulator, copper-sensing  transcriptional repressor |  |
| **Nickel resistance** | *nik*A | Nickel transport system substrate- binding protein |  |
|  | *nik*B | Nickel transport system permease protein |  |
|  | *nik*C | Nickel transport system permease protein |  |
|  | *nik*D | Nickel transport system ATP-binding protein |  |
|  | *nik*E | Nickel import ATP-binding protein NikE |  |
| **Magnesium-cobalt transport** | *tly*C | Magnesium and cobalt exporter, CNNM family |  |
| **Membrane remodeling** | | |  |
|  | *fab*B | 3-oxoacyl-[acyl-carrier-protein] synthase I |  |
|  | *fab*D | [acyl-carrier-protein] S-malonyltransferase |  |
|  | *fab*F | 3-oxoacyl-[acyl-carrier-protein] synthase II |  |
|  | *fab*G | 3-oxoacyl-[acyl-carrier protein] reductase |  |
|  | *fab*H | 3-oxoacyl-[acyl-carrier-protein] synthase III |  |
|  | *fab*I | enoyl-[acyl-carrier protein] reductase I |  |
|  | *fab*L | enoyl-[acyl-carrier protein] reductase III |  |
|  | *fab*Z | 3-hydroxyacyl-[acyl-carrier-protein] dehydratase |  |
|  | *des*A | Acyl-lipid omega-6 desaturase (Delta-12 desaturase |  |
|  | *des*K | Two-component system, NarL family, sensor histidine kinase DesK |  |
|  | *des*R | Two-component system, NarL family, response regulator DesR |  |

**Table S2.** List of compounds determined by GC-MS of strain MHN12 under control and salt stress conditions.

| **Compound Name** | **Probability % (Control)** | **Probability % (Stress)** |
| --- | --- | --- |
| n-Hexadecanoic acid (Palmitic acid) | 56.10% | 10.62% |
| Pyrrolo[1,2-a]pyrazine-1,4-dione, hexahydro-3-(2-methylpropyl) | 57.43% | 74.32% |
| Cyclopropanedodecanoic acid, 2-octyl-, methyl ester | 46.24% | Not Detected |
| Hexanoic acid, 2-methyl- | 49.16% | Not Detected |
| Pentanoic acid | 47.56 | 22.64% |
| Cyclopropanetetradecanoic acid, 2-octyl-, methyl ester | 46.24 | 27.05% |
| Tetradecanoic acid | 11.21 | Not Detected |
| Dodecanoic acid | 26.97 | Not Detected |
| Octadecenoic acid | 18.60 | Not Detected |
| Oleic Acid | Not Detected | 24.89 |
| Hexadecanol | Not Detected | 10.29 |
| Nonenoic acid | Not Detected | 15.27 |


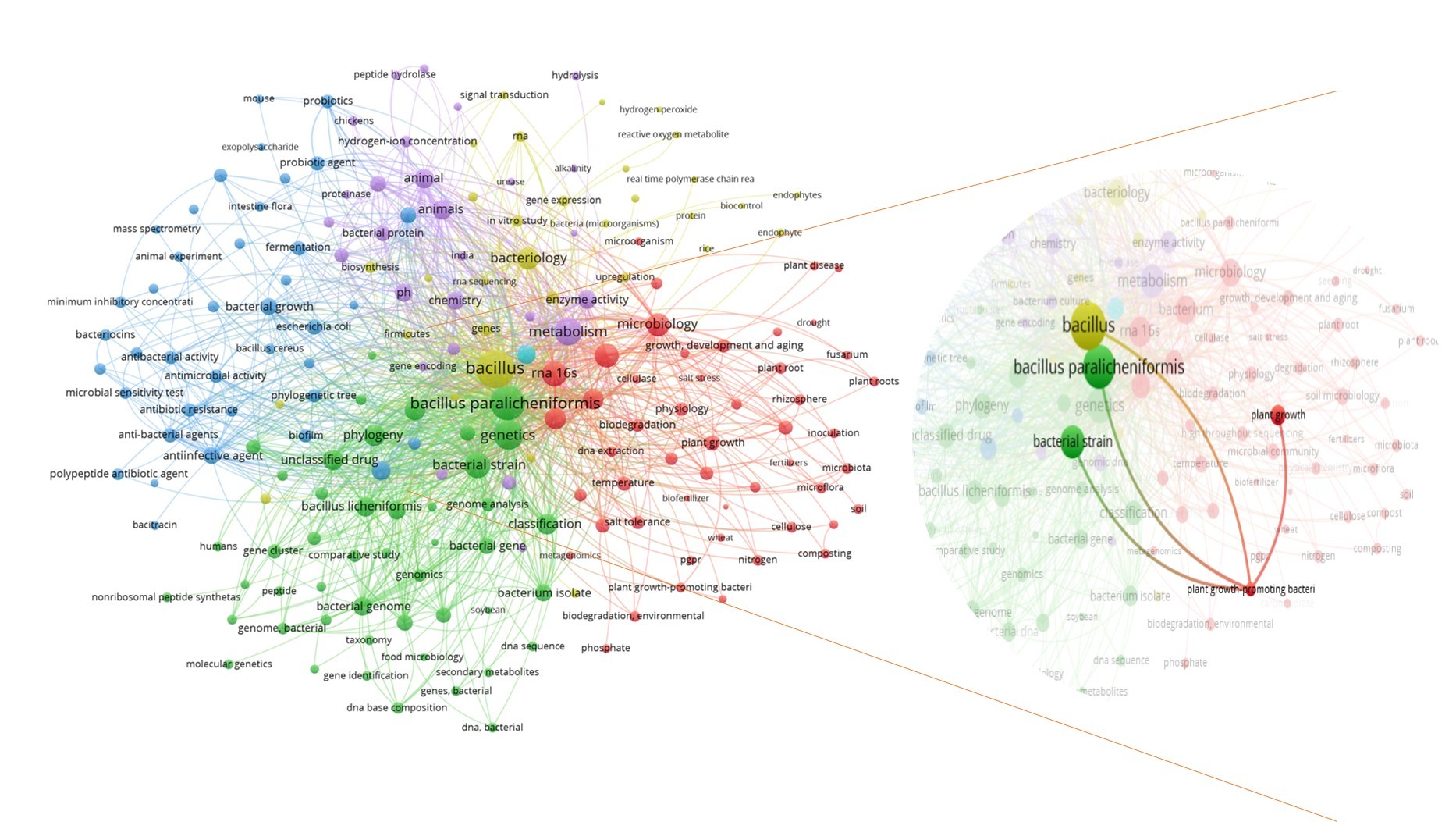


Figure 1. Bibliographic analysis of scientific research articles on *Bacillus paralicheniformis* generated using VOS viewer represents the existing literature predominantly targets its industrial uses, with limited focus on its role in salt tolerance and plant growth promotion.


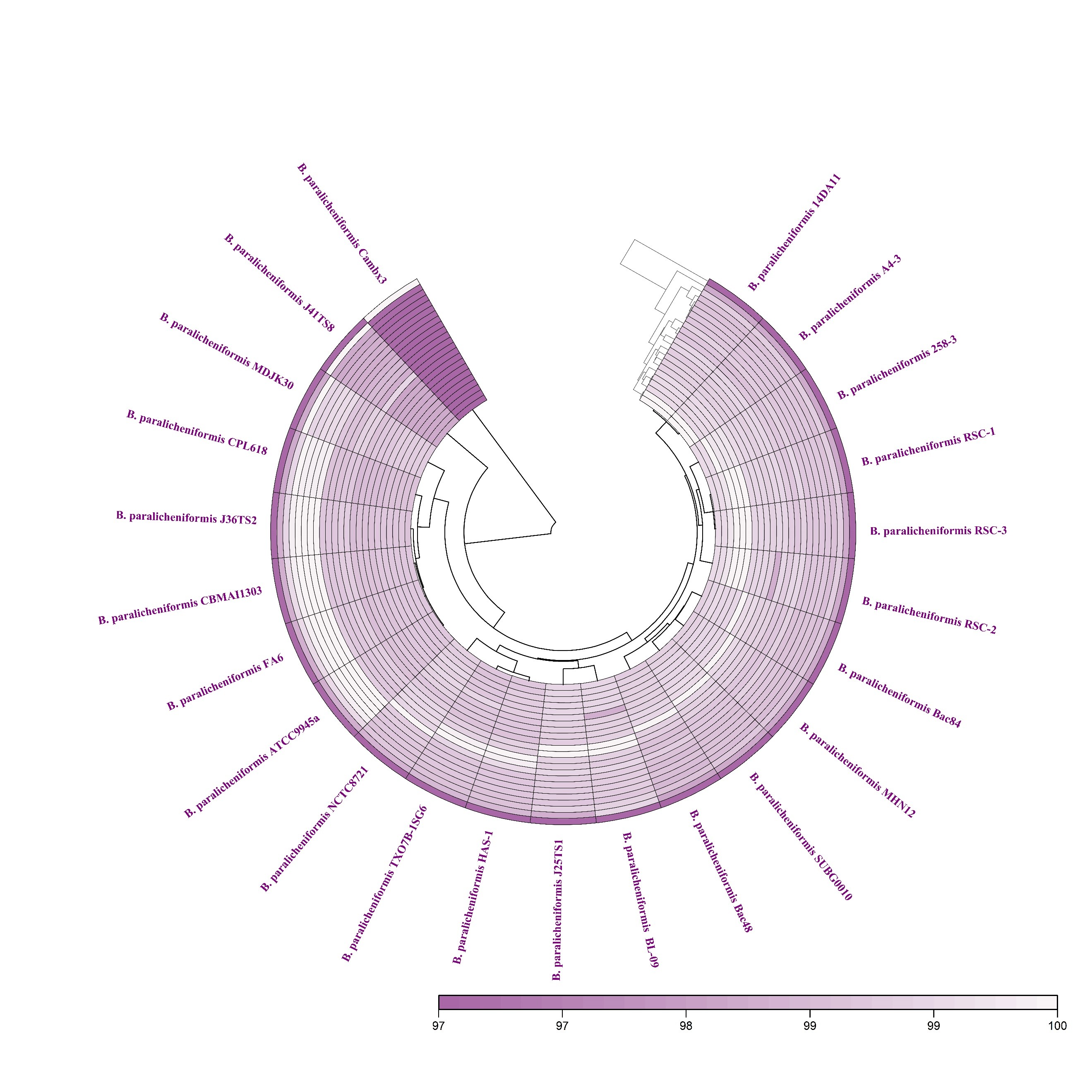


Figure 2. Heat map illustrating average nucleotide identity (ANI) between the strains under study.


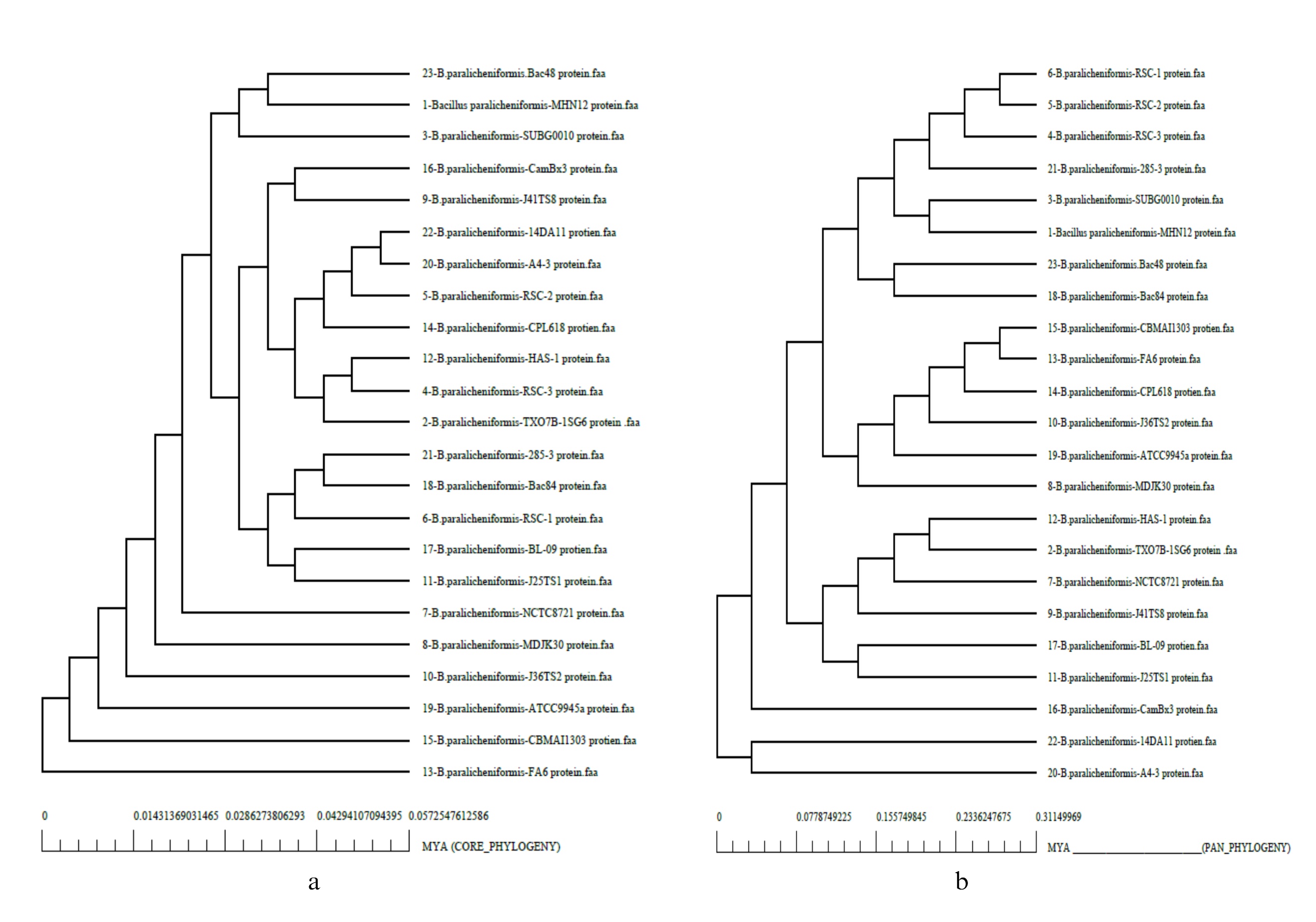


Figure 3. The phylogenetic relationships among *Bacillus paralicheniformis* strains based on the analysis of pan-genome.


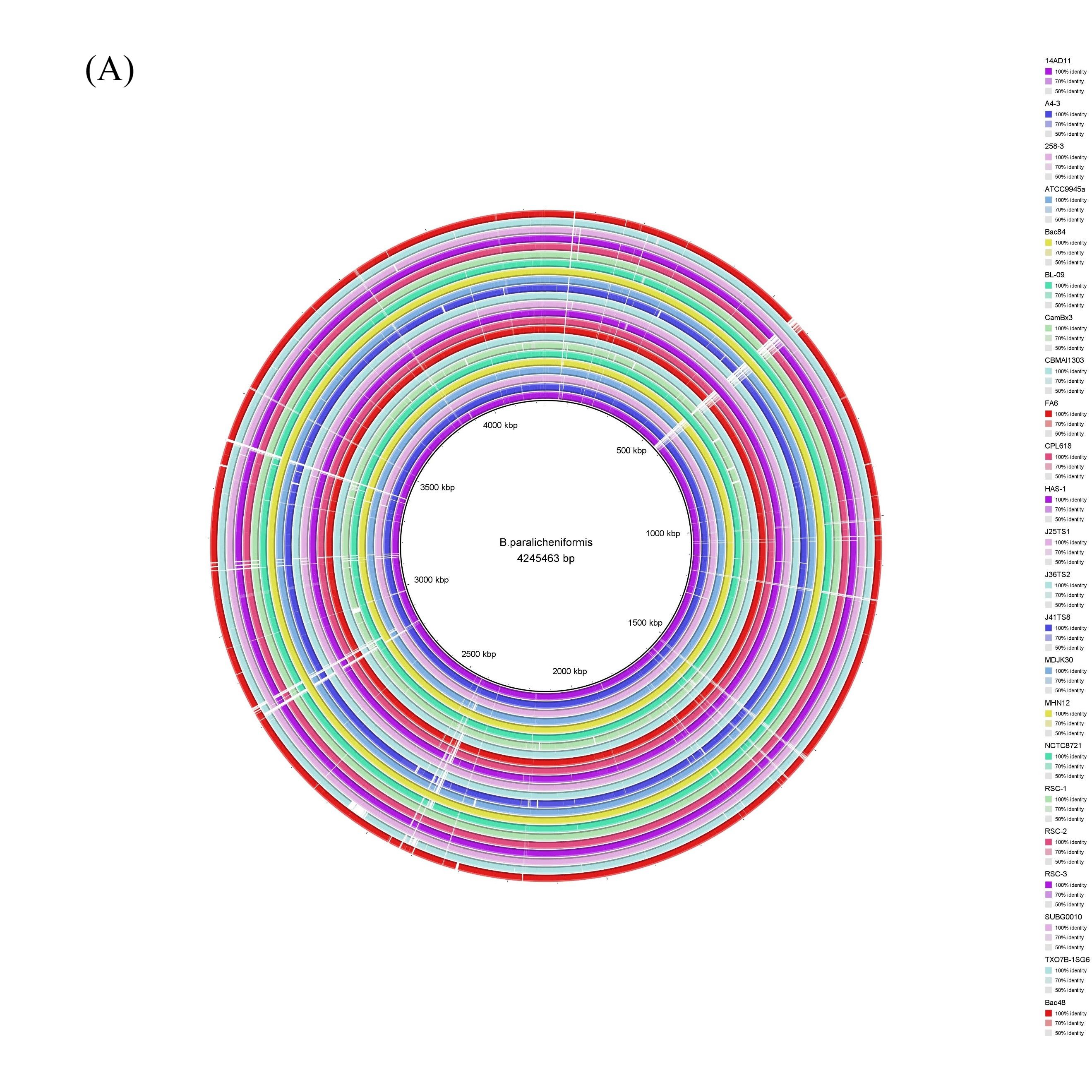


Figure 4. Genome comparisons of *Bacillus paralicheniformis* strains by BRIG using MHN12 as the reference strain.
